# The College of Nursing, Limited, and State Registration of Nurses

**Published:** 1916-11-25

**Authors:** Arthur Stanley, A. B. Baillie, E. Barton, J. Cantlie, R. Cox-Davies, A. C. Gibson, A. W. Gill, J. Glaister, A. Hughes, A. McIntosh, J. Melrose, Comyns Berkeley, W. Minet, E. W. Mowat, E. M. Musson, M. E. Ray, M. E. Sparshott, A. Lloyd Still, S. A. Swift, H. G. Turney, C. E. Vincent, Jane Walker, E. Cooper Perry

**Affiliations:** Chairman. 6 Vere Street, Cavendish Square, W.; (Hon. Treasurer), 6 Vere Street, Cavendish Square, W.; (Hon. Treasurer), 6 Vere Street, Cavendish Square, W.; (Hon. Treasurer), 6 Vere Street, Cavendish Square, W.; (Hon. Treasurer), 6 Vere Street, Cavendish Square, W.; (Hon. Treasurer), 6 Vere Street, Cavendish Square, W.; (Hon. Treasurer), 6 Vere Street, Cavendish Square, W.; (Hon. Treasurer), 6 Vere Street, Cavendish Square, W.; (Hon. Treasurer), 6 Vere Street, Cavendish Square, W.; (Hon. Treasurer), 6 Vere Street, Cavendish Square, W.; (Hon. Treasurer), 6 Vere Street, Cavendish Square, W.; (Hon. Treasurer), 6 Vere Street, Cavendish Square, W.; (Hon. Secretary), 6 Vere Street, Cavendish Square, W.; (Hon. Secretary), 6 Vere Street, Cavendish Square, W.; (Hon. Secretary), 6 Vere Street, Cavendish Square, W.; (Hon. Secretary), 6 Vere Street, Cavendish Square, W.; (Hon. Secretary), 6 Vere Street, Cavendish Square, W.; (Hon. Secretary), 6 Vere Street, Cavendish Square, W.; (Hon. Secretary), 6 Vere Street, Cavendish Square, W.; (Hon. Secretary), 6 Vere Street, Cavendish Square, W.; (Hon. Secretary), 6 Vere Street, Cavendish Square, W.; (Hon. Secretary), 6 Vere Street, Cavendish Square, W.; (Hon. Secretary), 6 Vere Street, Cavendish Square, W.


### The College of Nursing, Limited, and State Registration of Nurses.

*To the Editor of* The Hospital.

Sir,—We find that there is an impression that the action of the Central Committee for the State Registration of Nurses in breaking off negotiations with the College of Nursing will in some way hamper the activities of the Council, and delay their efforts to obtain State recognition for the nursing profession. This is not the fact.

The College stands for State registration, and intends to use every means in its power to obtain it. By far the most effective step that can be taken in this direction is for the nurses themselves to continue to put their names on the Register, so as to hasten the time when the scheme will be placed upon the democratic basis to which it is our desire to attain.

We mean also to endeavour to enlist such support from the public as will justify the Council in preparing complete plans, upon which operations can begin as soon as ever the war is over, for a building worthy to be the headquarters of the profession.

Such an Act and such a College will be a worthy memorial to the countless women who have served their country in our hospitals at home and abroad, often at the cost of health, and too often of life itself.

Unlike the Bill of 1914, which has already been before Parliament, and which establishes a Provisional Council, and keeps it in existence until the "Lord President of the Council certifies that the task of forming a Register of persons entitled to be registered is sufficiently advanced to admit of an election of direct representatives of registered nurses," the College Bill makes the existing Register of the College the first legal register, under which the nurses will proceed themselves to appoint their representatives upon the permanent Council so soon as the Provisional Council has finished its task of framing the rules under which the Act is to be carried out.

Under these circumstances we venture to hope that the nurses throughout the country, who have come forward with so much unanimity to support the College, will not relax their efforts; that they will interest their friends in it; and that they will sustain the Council in the prolonged struggle that lies before them in their endeavour to gain for nurses the legal recognition and professional status which they have so long desired.—We are, Sir, yours faithfully,

(Signed) Arthur Stanley, Chairman.

| | |
|---|---|
| A. B. Baillie, | W. Minet, |
| E. Barton, | E. W. Mowat, |
| J. Cantlie, | E. M. Musson, |
| R. Cox-Davies, | M. E. Ray, |
| A. C. Gibson, | M. E. Sparshott, |
| A. W. Gill, | A. Lloyd Still, |
| J. Glaister, | S. A. Swift, |
| A. Hughes, | H. G. Turney, |
| A. McIntosh, | C. E. Vincent, |
| J. Melrose, | Jane Walker, |
| Comyns Berkeley (Hon. Treasurer), | E. Cooper Perry (Hon. Secretary). |

6 Vere Street, Cavendish Square, W.,

November 22, 1916.

